# Ceramides during Pregnancy and Obstetrical Adverse Outcomes

**DOI:** 10.3390/metabo13111136

**Published:** 2023-11-08

**Authors:** Maria Lantzanaki, Theofanis Vavilis, Vikentia C. Harizopoulou, Helen Bili, Dimitrios G. Goulis, Dimitrios Vavilis

**Affiliations:** 1 1st Department of Obstetrics and Gynecology, Medical School, Aristotle University of Thessaloniki, 541 24 Thessaloniki, Greece; vikentiaha@yahoo.com (V.C.H.); helbil@auth.gr (H.B.); dgg@auth.gr (D.G.G.); vavilis.dimitrios@ucy.ac.cy (D.V.); 2 Department of Dentistry, School of Medicine, European University of Cyprus, Nicosia 2404, Cyprus; thvavilis@gmail.com; 3 Laboratory of Medical Biology and Genetics, School of Medicine, Aristotle University of Thessaloniki, 541 24 Thessaloniki, Greece; 4 Medical School, University of Cyprus, Nicosia 1678, Cyprus

**Keywords:** ceramides, obstetrics, pregnancy

## Abstract

Ceramides are a group of sphingolipids located in the external plasma membrane layer and act as messengers in cellular pathways such as inflammatory processes and apoptosis. Plasma ceramides are biomarkers of cardiovascular disease, type 2 diabetes mellitus, Alzheimer’s disease, various autoimmune conditions and cancer. During pregnancy, ceramides play an important role as stress mediators, especially during implantation, delivery and lactation. Based on the current literature, plasma ceramides could be potential biomarkers of obstetrical adverse outcomes, although their role in metabolic pathways under such conditions remains unclear. This review aims to present current studies that examine the role of ceramides during pregnancy and obstetrical adverse outcomes, such as pre-eclampsia, gestational diabetes mellitus and other complications.

## 1. Introduction

Ceramides are a diverse family of sphingolipids in mammalian cell membranes synthesized by at least six ceramide synthases [1]. From a structural point of view, they consist of an 18-carbon-amino-alcohol backbone, usually sphingosine, linked via an amide bond to a fatty acid chain [2]. The latter can vary in terms of saturation and length, encompassing medium (12–13), long (16–18), very long (20–24), and ultra-long (>26) carbon atoms [3]. The number of structurally unique ceramides exceeds 1500; novel molecules are discovered due to the continuous development of analytic techniques [4,5]. Despite their diversity, ceramides constitute less than 1% of cell membrane lipids. Their concentrations can be modified by various physiological and pathophysiological stimuli [6].

**Figure 1:**
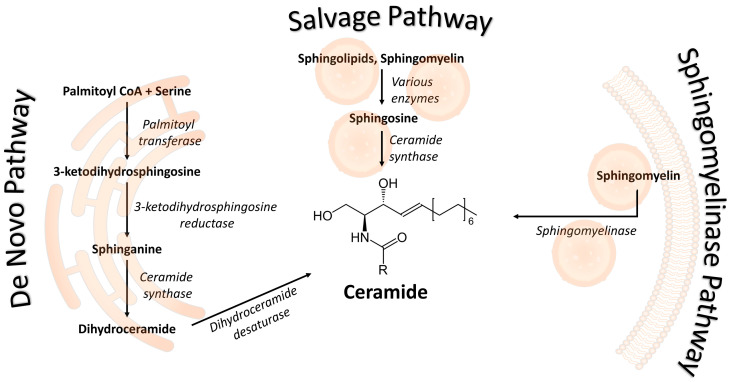
Ceramide Synthesis Pathways: This figure provides an overview of the three pathways of ceramide synthesis. The first pathway, (de novo), operates within the endoplasmic reticulum. The second pathway, (salvage), relies on lysosomes. The third pathway, (sphingomyelinase), can occur with the participation of both cellular membranes and lysosomes. Enzymes associated with each step are denoted in *italics*.

Three ceramide biosynthesis pathways have been identified: an anabolic (de novo), a mixed recycling (salvage) and a catabolic (sphingomyelinase) pathway (Figure 1) [7]. The de novo pathway, as the name implies, synthesizes ceramides from simpler molecules through a multi-step procedure in the endoplasmatic reticulum [8,9,10,11,12]. The synthesis starts with a condensation of palmitoyl-CoA with the amino acid serine aided by the enzyme serine palmitoyltransferase to yield 3-ketodihydrosphingosine. This product is converted to sphinganine by 3-ketodihydrosphingosine reductase, followed by acylation via ceramide synthase to yield dihydroceramide. Final conversion to ceramides takes place through the catalytic desaturation by dihydroceramide desaturase. Ceramides can be further modified in the Golgi apparatus to produce an array of complex sphingolipids, such as glycosphingolipids and sphingomyelin [9]. The salvage pathway recycles ceramides from complex sphingolipids and sphingomyelin in lysosomes/late endosomes. This pathway produces sphingosine as an intermediate converted to ceramides [13]. Finally, the sphingomyelinase pathway produces ceramides and phosphocholine via direct sphingomyelin hydrolysis [14]. This conversion can occur in plasma membrane or lysosomes and is implicated in signaling cascades [15]. Ceramides are also available to cells indirectly through diet: eggs, dairy products, and soy are rich in sphingolipids, which can be intracellularly converted into ceramides [16]. Several xenobiotics can affect ceramide biosynthesis, such as perfluorochemicals (PFCs) and phthalates (DAP). More research is needed to define the effects of environmental exposures to lipid metabolism [17].

Ceramides play a multi-faceted role in the cell. They constitute an essential cell membrane component, provide structural integrity, and are biologically active [1]. On the one hand, ceramides act as secondary messengers, modulating the activity of various phosphatases and kinases. On the other hand, they directly influence gene transcription by affecting transcription factors [18]. As such, ceramides are implicated in various physiological and pathophysiological responses of the cells, such as programmed cell death, differentiation, proliferation, cell cycle arrest, inflammation, and stress response [1,18]. These responses render them relevant to many diseases. Ceramides potentially contribute to the pathogenesis of various autoimmune conditions, including rheumatic diseases and psoriasis, and cardiovascular and metabolic disorders, such as insulin resistance, diabetes, and gestational diabetes [2,6,19,20,21,22,23,24,25,26]. Emerging evidence suggests an association with cancer through epigenetic mechanisms and psychiatric disorders [27,28]. Given the above, ceramides appear attractive and promising novel clinical biomarkers for various diseases, assisting in their diagnosis and treatment [1,29]. In this review, we will describe the role of ceramides during pregnancy and their association with major obstetrical adverse outcomes, such as pre-eclampsia and gestational diabetes mellitus.

## 2. Relevant Sections

### 2.1. Ceramides during Pregnancy

As mediators of stress signals and pro-inflammatory responses, ceramides play important roles in gestation, an inflammatory process with distinct phases at blastocyst implantation and delivery. In this context, Signorelli et al. [30] proposed ceramide as a therapeutic target in inflammatory labor complications based on differences in total ceramide and serine-palmitoyl transferase between vaginal and cesarean deliveries. Similar patterns of ceramide accumulation in the maternal and cord plasma were observed in a pilot comparison between laboring and non-laboring women [31]. Based on comprehensive profiling of numerous metabolites, the endocannabinoid/ceramide/sphingolipid pathway has the potential to stimulate labor [31]. In addition, ceramides play signaling roles in various reproductive tissues [32] and mediate vascular function during healthy pregnancy [33].

Longitudinal studies are needed to understand the role of ceramides in pregnancy, but these are scarce. One study in cows followed lipid accumulation in the transition from gestation to lactation and showed that ceramides and glycosylated ceramides accumulate during the transition, while plasma sphingomyelin, a potential source of ceramides, reaches a nadir at labor. This and other findings were interpreted as ceramides being an intrinsic mediator of adaptive metabolism that accompanies the onset of lactation and facilitates milk synthesis [34]. In humans, the only longitudinal study followed babies of obese mothers at delivery and four months after. The study showed that the nutritional status of the mother during pregnancy affects the children’s ceramide profile in the long term [35]. The alteration of ceramide concentrations as a function of gestational age in full-term uncomplicated pregnancies and healthy donor controls was simulated with regression analysis while developing a liquid chromatography with tandem mass spectrometry (LC-MS/MS) assay. Besides quantification along the mid-to-late gestation weeks in human serum samples, the study identified four novel endogenous species of ceramides [(d18:1/17:0), (d18:1/20:1), (d18:1/21:0), (d18:1/24:2)], revealing that novel ceramides remain to be discovered [36]. A recent study shows that ceramides are elevated during pregnancy compared with postpartum. The actions of ceramides and SP1 on endothelial function is crucial for an uncomplicated pregnancy to take place [37].

From the above, it is apparent that sphingolipid metabolism is finely regulated during gestation and is tissue-specific. This effect has been shown in mice at the maternal-embryonic interface [38]. It has been proposed that ceramides in fetal blood are a fetal product. In contrast, the absence of correlation between the concentration of fatty acid-containing ceramides in the umbilical cord and the maternal plasma indicated that the rate of fatty acid metabolism within the placenta is high [35,39].

There are no studies concerning the correlation of diet during pregnancy with ceramide concentrations. Nevertheless, a correlation between obesity and ceramide concentrations in non-pregnant populations has been reported [40]. More studies are needed to elucidate the interaction between adipose tissue and ceramide concentrations.

### 2.2. Ceramides in Preeclampsia

Pre-eclampsia (PE) is a multi-system pregnancy disorder affecting up to 8% of pregnant women and is associated with a high risk of preterm births [41,42]. It is the leading cause of maternal mortality worldwide [43,44], increasing the risk of developing cardiovascular and renal disease long after pregnancy [45,46]. Patients who develop preeclampsia have placental abnormalities from the first trimester of pregnancy such as spiral artery remodeling and hypoxia [47]. Hemochoridal placentation brings in contact maternal vessels with trophoblast cells; this materno–fetal interaction initiates an immune response resulting in abnormal trophoblast invasion. These mechanisms contribute to the establishment of a proinflammatory environment [48]. Asymptomatic in the first trimester of pregnancy, it is manifested in the second half by the sudden onset of maternal hypertension; coexisting symptoms include proteinuria and generalized edema [49]. Besides preterm delivery, treatment interventions include steroids, magnesium, antihypertensive therapy, and aspirin, established as standard preventive care for at-risk women [50]. Markers of PE adopted in the clinical practice are indicators of endothelial damage or hypoxia since PE implies low oxygen availability for the fetus and increased trophoblast autophagy and apoptosis for the mother, causing systemic vascular endothelial injury. Nevertheless, efficient prognosis and treatment of PE can only be achieved through early diagnosis, ideally in the first trimester of pregnancy. The task remains challenging despite several studies ranging from urine proteomics, metabolomics, and epigenomics to predictive algorithms that combine maternal risk factors or personalized medicine protocols based on combinations of phase IIa clinical omics data [51,52,53,54]. For instance, a maternal sera test based on the soluble vascular endothelial growth factor receptor-1 (sFlt-1)/placental growth factor (PlGF) ratio (i.e., the imbalance between two placental-derived angiogenic and anti-angiogenic factors), differentiates PE from uncomplicated pregnancies, but only in the third trimester of pregnancy [55,56].

Ceramides emerge as a promising diagnostic marker and therapeutic target of PE. Increasing attention is given to lipid imbalances. The roles of lipid metabolism in the pathophysiology of adverse outcome pregnancies have been suspected for a long time, but not studied in large cohorts [57]. Lipidomics, alone or combined with expression, imaging, and bioinformatic approaches, is the method of choice to understand the changes occurring during PE-complicated gestation. One large multiethnic cohort study identified Cer-NS d30:1 as a potential maternal sera biomarker of severe PE [58]. The advantage of the study was that it identified molecules uniquely associated with PE against a series of clinical confounders; however, since samples were taken at delivery, the ceramide suitability for early diagnosis remains to be investigated. Two ceramides have been proposed as early diagnostic markers of PE. The first is Cer 14, the concentrations of which (along with those of SM 16 and SM 18) were decreased in PE maternal plasmas compared with controls in the first trimester of pregnancy [59]. The study is one of the rare stratified cross-gestational analyses of ceramides in PE, but the sample size was small, awaiting confirmation from larger studies. The second is an odd-chain ceramide (d18:1/25:0). Although only suitable for mid-gestation PE diagnosis, when incorporated in a ratio with leptin [Lep/Cer (d18:1/25:0)], it performs better than the sFlt-1/PlGF ratio for early PE prediction [60].

The search for ceramides suitable for early diagnosis relies on a comprehensive understanding of their regulation during PE and gestation, which remains controversial. Lipidomic studies are heterogeneous regarding PE characteristics (mild, severe, comorbidities, treatment), tissues (blood, placenta, fetal), and gestational stage (Table 1). Sampling is technically easy during late gestation and delivery, allowing a more complete overview. In the maternal blood, ceramides Cer 16, Cer 18, and Cer 20 consistently increased instances of PE [61,62,63]. It has been proposed that the accumulation of ceramides in PE-complicated pregnancies is of placental origin [63], although alternative origins, such as liver injury or platelet activation, have been proposed [61]. Among ceramides, the levels of which are increased in the maternal circulation, C16 and C18 also accumulate in the placental mitochondria, while D-e-C18:0 is increased in aspirin-treated women with severe PE [63,64,65]. Consistent with cell-dependent regulation of sphingolipid metabolism [9], C20:0-Cer is reduced in the placenta, compared with chorionic arteries and isolated endothelial cells from uncomplicated pregnancies [66] The same pattern of decrease is observed in the two studies conducted in the fetus [67,68]; PE umbilical cord veins showed a decrease in total ceramides and an increase in sphingosine-1-phosphate (S1P) at birth [69]. Some of these changes are linked to signaling deregulation. Ceramides act as bioactive molecules that transmit signals in the cells, while they constitute structural components of lipid rafts that host several receptors involved in cell-to-cell communications [67,70]. Elevated concentrations of long-chain (C16:0–C24:0) pro-apoptotic ceramides, along with reduced concentrations of circulating angiogenic S1P, indicate an impairment of the sphingolipid S1P:CER rheostat in the placenta [32,63]. The accumulation of ceramides in this organ may not only contribute to the excessive cell death observed in PE, but was proposed to promote necroptosis, disrupting the normal trophoblast fusion [71]. At the surface of the placenta and in direct contact with maternal blood, the multinucleated cell layer of the syncytiotrophoblast experiences aberrant cell death associated with ceramides. A metabolomic study of villous extract cultures from placenta treated with the murine monoclonal antiphospholipid antibody ID2 proposed that maternal antiphospholipid antibodies induce ceramide dysregulation early during gestation [72].

With improvements in lipidomics continuously unlocking the potential of these technologies for comprehensive tissue profiling, additional aspects of ceramide roles in PE are expected to be discovered. The studies are limited in sample size and inclusivity. Notably, the average size rarely exceeds 20, which does not allow for robust conclusions, especially in a comparative context. Moreover, although ethnicity has been identified as a confounding factor for PE [58], cohorts are rarely multiethnic, in line with disparities in PE management and outcomes [73].

### 2.3. Ceramides in Gestational Diabetes Mellitus

Defined as a decreased response of the peripheral tissues to insulin action, insulin resistance (IR) is a multi-faceted disorder triggered by obesity and consumption of a high-fat diet. Numerous studies in humans, rodents, and cell cultures have established participation of ceramides in the development of IR. Ceramides antagonize insulin signaling by interfering with glucose uptake and impairing the storage of nutrients, while they inhibit oxidation, stimulate fatty acid uptake, and enhance cell death. Ceramide accumulation in skeletal and adipose tissue exerts the above effects by inhibiting signal transmission and activating pro-inflammatory cytokines and other regulatory molecules [70]. Accumulating ceramides in skeletal muscles and the liver appear to underlie IR related to hypoxia-induced developmental fetal delays in rats, although the role of pro-inflammatory signaling has been questioned [74].

Besides its association with pathologies, such as obesity and type-2 diabetes mellitus (T2DM), IR has been observed as a metabolic adaptation in the transition from late pregnancy to early lactation. Female adipose tissue metabolism changes from lipogenic to lipolytic at this stage to ensure adequate nutrition, development and milk synthesis for the growing fetus. The role of ceramides in this transition has been poorly investigated. In pregnant women, long- and very long-chain ceramides (18:0, 22:0, and 24:l) commonly associated with the development of T2DM were positively correlated with the triglyceride index, a proxy of insulin resistance that reaches its highest values during pregnancy [75]. Accumulation of ceramide was also observed in a longitudinal study of dairy cattle, an increase representing a homeorhetic adaptation, since it affects lean and overweight cattle [34]. By contrast, reduced ceramide concentrations carrying C22:0, C23:0, and C24:0 saturated fatty acyl chains were reported in the offspring of obese mothers [35].

Beyond the inherent IR associated with pregnancy, gestational diabetes mellitus (GDM) is a common complication characterized by glucose intolerance and hyperglycemia [76]. As its pathophysiology arises from IR and has commonalities with T2DM, ceramides play important, yet unclear roles in GDM. Changes in specific ceramide concentrations (increased 18:0, 18:1 and decreased ceramide 24:0) in early pregnancy increase the risk of GDM [77]. Decreased ceramide 24:0 concentrations were reported in the only study in full-term pregnancies complicated by GDM [26]. Although epidemiological studies have suggested that lipid metabolic profiles differ between women with and without GDM [78], most suffer from reverse causation bias and rarely study the first trimester of pregnancy, which is crucial for the development of GDM. A recent birth-cohort lipidomic study addressed the above shortcomings. The study identified two ceramides, monohexosyl 18:0, and dihexosyl 24:1, among 10 lipid molecules associated with GDM, independently of confounders [79]. This was the first report that identified these two ceramides as negatively associated with GDM. As their addition in a predictive GDM model improved its discriminatory power, these molecules appear as novel early predictors of GDM. The role of ceramides in early pregnancy was confirmed by a study of a smaller sample size that identified a different biomarker, C18:1-Cer. In partial agreement with Liu et al. [77], the ceramide showed higher concentrations in pregnant women later diagnosed with GDM than those with normal glucose tolerance [80]. Another study of 1998 women found that only ceramide 24:0 in early pregnancy had a positive predictive value for GDM [81].

GDM is conventionally treated with insulin. Nevertheless, a study demonstrated disrupted trophoblast function in insulin-treated individuals. Through immunohistochemistry, the study established a link between ceramide accumulation and hyperosmolar stress in the placenta and showed that insulin and ceramide may elicit adverse mitochondrial anomalies [82].

### 2.4. Ceramides in Other Pregnancy Complications

Besides PE and GDM, ceramides have been associated with pregnancy complications. An example is the identification of glucoside ceramides as sensitive and specific indices of chorioamnionitis. The latter is often associated with preterm labor. Accumulation of the ceramide was found in granulocytes, fetal membranes neutrophils, and corresponding amniotic fluids, but was acknowledged only to reflect the activity of inflammatory cells [83]. Later, the mechanism by which ceramides participate in the inflammatory process was revealed to be direct, acting as a second messenger to induce prostaglandin production in amnion and decidual cells [84]. Ceramide concentrations are higher in women with preterm birth and chorioamnionitis compared with those with preterm birth and no signs of chorioamnionitis [83]. It has not been elucidated whether the inflammation results in an elevation of ceramide concentrations, or vice versa [85].

Studies highlighting changes in ceramide concentrations in preterm labor indicate that different types might be involved in its pathogenesis at distinct gestation stages. At 20 gestational weeks, reduced expression of ceramides CerP(44:1) and galactosyl-Cer(42:2) was observed in association with spontaneous preterm birth [86]. At the same stage, recurrent pregnancy losses have been associated with Cer(d18:0/16), the latter being downregulated in a metabolomic study of the decidua [87]. At mid-gestation, ceramide d40:2 was identified as a part of the dyslipidemic profile of underweight women [88], while after 32 gestational weeks, elevated plasma C16-Cer concentrations were identified as the best predictor of preterm labor [85].

The mechanism by which ceramides and, in general, sphingolipids exert their effects in preterm labor is unclear, but the most plausible hypothesis is that they activate pro-inflammatory transcription factors involved in uterine contractions and cervical ripening. Ceramides may further contribute to preterm birth by affecting liver functions, as indicated in two lipidomic studies focusing on intrahepatic cholestasis of pregnancy (ICP), a liver dysfunction that increases the risk for preterm birth. In one study, ceramides 18:1/22:0 and 18:1/24:0 were downregulated in mid and high-severity ICP compared with uncomplicated pregnancies [89]. In contrast, Cer C16 and C18 were increased in ICP pregnancies compared with uncomplicated ones and were further affected by treatment with ursodeoxycholic acid [90]. Both studies suffered from small size and confounding factors; thus, the changes in ceramides and other sphingolipids could not be safely associated with the onset of ICP.

The increased risk of adverse pregnancy outcomes, including miscarriage and low birth weight, indirectly relates to sphingolipid metabolism, through polycystic ovary syndrome (PCOS). Two types of ceramides, hexosylceramide HexCer (d18:2/24:0) and dihydroceramide Cer (d18:0/24:1), were reported among the best indicators of low birth rate (up to 10% lower) in newborns of women with PCOS compared with their matching controls [91]. Moreover, ceramide alterations during pregnancy disorders are passed to the embryos. In Down syndrome, the pathophysiology relates to impaired myelination/demyelination of brain neurons; ceramide concentrations were altered in maternal plasma and amniotic fluid in pregnancies with fetal chromosomal abnormalities [62]. Similarly, long-term alterations in ceramide concentrations were detected in the plasma of offspring of obese mothers [35].

Studies highlighting ceramides’ roles in other pregnancy complications have made a case for extending clinical trials on substances targeting sphingolipids. Initially part of anti-inflammatory strategies, there is growing interest in using them as therapeutic targets of preterm labor and ICP [85].

## 3. Discussion

Ceramides play an important role throughout gestation and labor. A higher concetration is observed during natural birth compared to caesarian section due to the inflammatory process. In addition, ceramides may stimulate labor. There are few data about ceramide concentrations during pregnancy and the impact of diet on their concentrations.

Most research on pregnancy and adverse obstetrical outcomes has been conducted for preeclampsia. Biomarkers of preeclampsia are related to hypoxia and endothelial damage. Although the exact metabolic pathway is unknown, abnormal placental angiogenesis during preeclampsia seems to have an impact on ceramide production. The elevation of ceramide concentration in preeclampsia is caused not only from placental abnormalities, but also from liver injury and platelet activation. Data from current research, regarding the correlation of ceramides alterations with preeclampsia, are conflicting. Long chain ceramides are the most affected.

Ceramides have also been studied in gestational diabetes mellitus. It is known that ceramides are altered in individuals with obesity, insulin resistance and type 2 diabetes mellitus. Long chain ceramides, especially Cer 18 and Cer 24, were found to be related to gestational diabetes mellitus.

Other obstetrical adverse outcomes, such as preterm labor, were related to alterations of ceramide concentrations in a few studies. Chorioamnionitis, an inflammation of the membranes and chorion of the placenta, is a major cause of preterm labor and ceramides are elevated due to the inflammatory process. Another explanation of ceramide alterations in preterm labor are pre-inflammatory agents deriving from uterine contractions and cervical ripening. Few data exist on intrahepatic cholestasis of pregnancy and ceramides.

## 4. Conclusions

Ceramides have been studied in various health conditions due to their role in signaling pathways and their potential use as biomarkers. Pregnancy and obstetrical adverse effects are complex situations in which ceramides may be involved. Ceramides are mediators of stress and seem to play a role, especially during implantation, labor and lactation. The current literature about obstetrical adverse effects includes pre-eclampsia, gestational diabetes mellitus, preterm birth, chorioamnionitis, intrahepatic cholestasis of pregnancy and fetal chromosomal abnormalities. Only a deeper comprehension of their role in uncomplicated pregnancy and during pregnancy adverse outcomes will allow possible use of ceramides in clinical practice.

## 5. Future Directions

Lipids seem to play a crucial role throughout pregnancy and obstetrical adverse outcomes. First trimester biomarkers could improve prognosis and therapeutic strategies. Ceramides are potential and promising biomarkers of such complications as preeclampsia and gestational diabetes mellitus. The current literature on alteration of ceramides is limited and results are conflicting. More research in large cohorts is needed to define their role in metabolic pathways and clinical use as pregnancy biomarkers.

## Figures and Tables

**Table 1:** Studies investigating ceramide content in relation to PE.

Tissue	Study	Pathology	Ceramide Concentration Changes	Gestational Stage	Group Size (PE vs. Control)	Method
**Embryo**						
Umbilical cord: veins	Romanowicz, 2010	PE	Decrease (total SAFA, MUFA, PUFA)	late **	10 vs. 10	TLC-HPLC
Umbilical cord: Wharton jelly	Romanowicz, 2010	PE	Increase (total, SAFA, MUFA)	late	10 vs. 11	TLC-HPLC
**Placenta**						
Placenta	Amraoui, 2020; Walsh, 2020; Melland-Smith, 2015	PE + HELLP; PE + aspirin; PE	No difference; Increase: D-e-C18:0 (severe risk + aspirin); Increase (16–18–20–24)	late; late; late	11 vs. 24/13; 13 vs. 14; 45 vs. 40	LC-MS; UPLC ESI-MS/MS; LC-MS/MS
Mitochondria	Ausman, 2018	PE	Increase: 16, 18	late	4 vs. 4	LC-MS/MS
Arteries and endothelial cells	Delgaudio, 2020	PE	Decrease: 20	late	8 vs. 10	LC-MS/MS
Syncytiotrophoblast microvesicles	Nbaig, 2013; Pantham, 2015	PE and RM; PE model *	No difference; Increase 18:1/24:1) in first trimester and ceramide-1-phosphate (d18:1/12:0) in third trimester	late; early and late	6/9 vs. 6/9; 90 early and 54 late	LC-MS; UHPLC-MS
**Mother**						
Plasma	Dobierzewska, 2017; Amraoui, 2020; Charkiewicz, 2017	PE; PE + HELLP; PE mild	Decrease: 24 in third, 14 in first trimester; Increase (total); increase: 16, 18, 18:1, 20, 22, 24	all; late; late	7 vs. 7; 11 vs. 24/13; 21 vs. 36;	HPLC-ESI-MS/MS; LC-MS; UHPLC/MS/MS
Serum	Huang, 2021; He, 2021; Melland-Smith, 2015	PE; PE severe; PE	Decrease: d18:1/25:0 in second trimester Decrease: Cer-NS d30:1; Increase: 16–18–20–24	all; late; late	20 vs. 20; 44 vs. 20; 45 vs. 40	LC-MS/MS; LC-MS/MS; LC-MS/MS

One study [71] was excluded because it did not report ceramide concentrations. * Treatment with an antiphospholipid antibody associated with high risk of PE, indirect. ** Third trimester of pregnancy or delivery/newborns ESI: electrospray ionization; HELLP: hemolysis, elevated liver (enzymes), low platelets; HPLC: high-performance liquid chromatography; LC: liquid chromatography; MS: mass spectrometry; MUFA: monounsaturated fatty acid; PE: preeclampsia; PUFA: polyunsaturated fatty acid; RM: ruptured membranes; SAFA: saturated fatty acid; TLC: thin-layer chromatography; UHPLC: ultra-high-performance liquid chromatography; UPLC: ultra-performance liquid chromatography.
